# Supplementary material for: Somatic whole genome dynamics of precancer in Barrett’s esophagus reveals features associated with disease progression
Source: Nat Commun. 2022 Apr 28;13:2300. doi: 10.1038/s41467-022-29767-7 (PMC9050715; doi:10.1038/s41467-022-29767-7)
Supplement: Supplementary file 1 — Supplementary Information [file 41467_2022_29767_MOESM1_ESM.pdf]

Supplementary Fig. 1

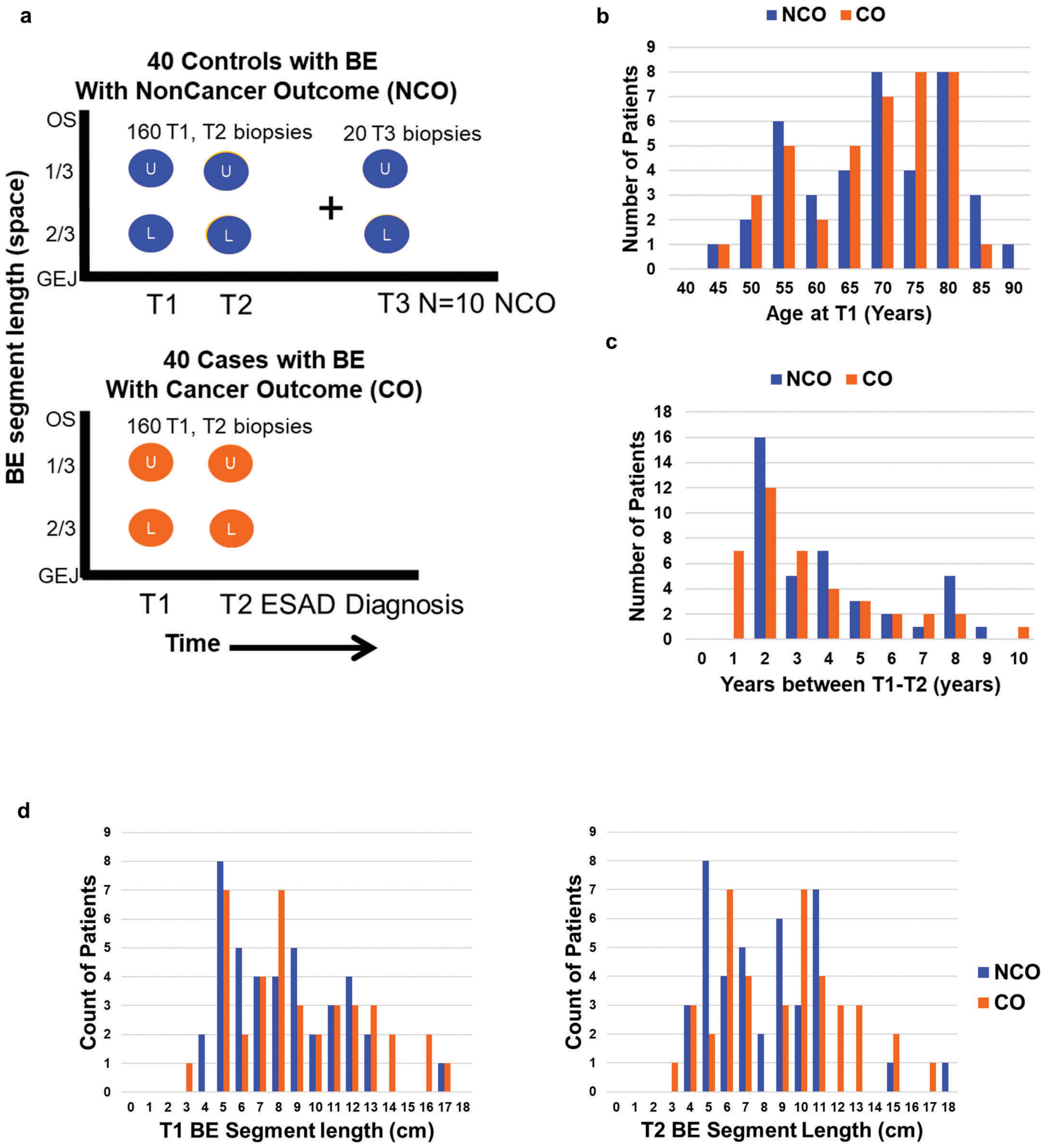

Supplementary Fig. 1. a, 40 NCO controls (top panel, blue) who remained cancer free during endoscopic surveillance were assessed at two timepoints (T1, T2), with two samples per time point at physical locations including one in the upper (U) proximal ⅓ of the annotated BE segment between the ora serrata (OS) and the gastroesophageal junction (GEJ), and one in the lower (L) distal ⅔. An additional long follow-up time point with the same sampling strategy was collected for 10 of these NCO. 40 CO cases (bottom panel, orange) who progressed to an endoscopically detected, incident ESAD similarly assessed and sampled as the NCO. **b,** Patient age distribution at T1 time point. Age numbers indicate interval from the previous age number (i.e., 45 includes patients aged 40 to 45). NCO and CO were matched on patient age at T1. **c,** Distribution of years between T1 and T2 timepoints. **d,** BE segment length (gastroesophageal junction cm - ora serrata cm) in cm at the T1 time point (left panel) and the T2 time point (right panel).

Supplementary Fig. 2

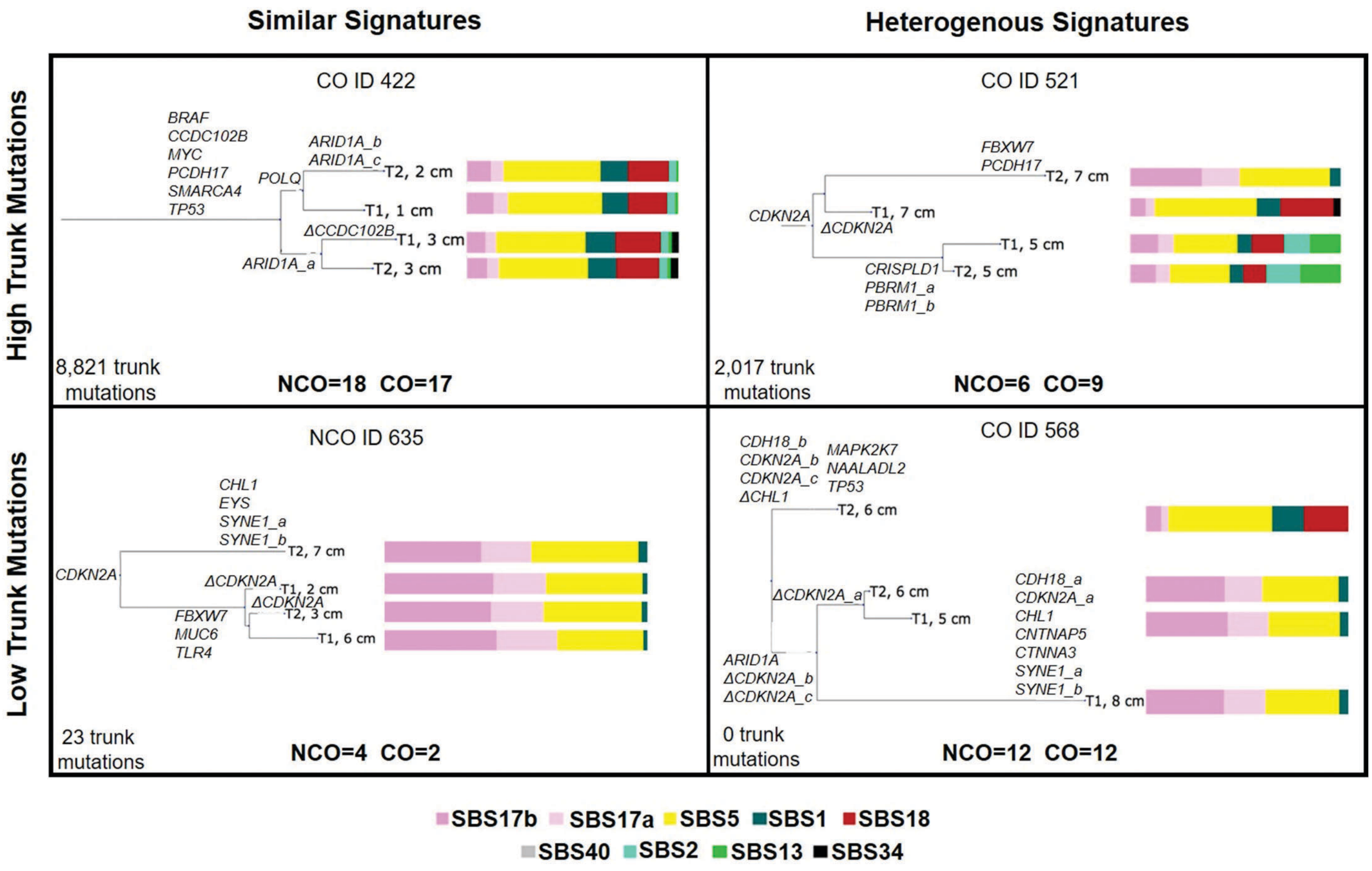

Supplementary Fig. 2. Top panels show phylogenies and proportion of each mutation signature per sample in two CO cases with high levels of trunk mutations (≧ 289 somatic SNVs or indels shared between all biopsies). Bottom panels show one NCO and one CO with low trunk mutations (<289 SNVs or indels shared by all biopsies). Phylogeny tips are annotated with time-point (T1=Timepoint 1, T2=Timepoint 2) and biopsy centimeter (cm) distance from the gastroesophageal junction. ESAD genes of interest with functional mutations are annotated on the phylogeny. Left panels show two examples with similar mutation signatures (using cosine similarity coefficient) across all biopsies. Right panels show two examples with heterogeneous mutation signatures. The total number of NCO and CO in each category is noted at the bottom of each box within the panel. The legend for mutation signature is shown beneath the figure.

Supplementary Fig. 3

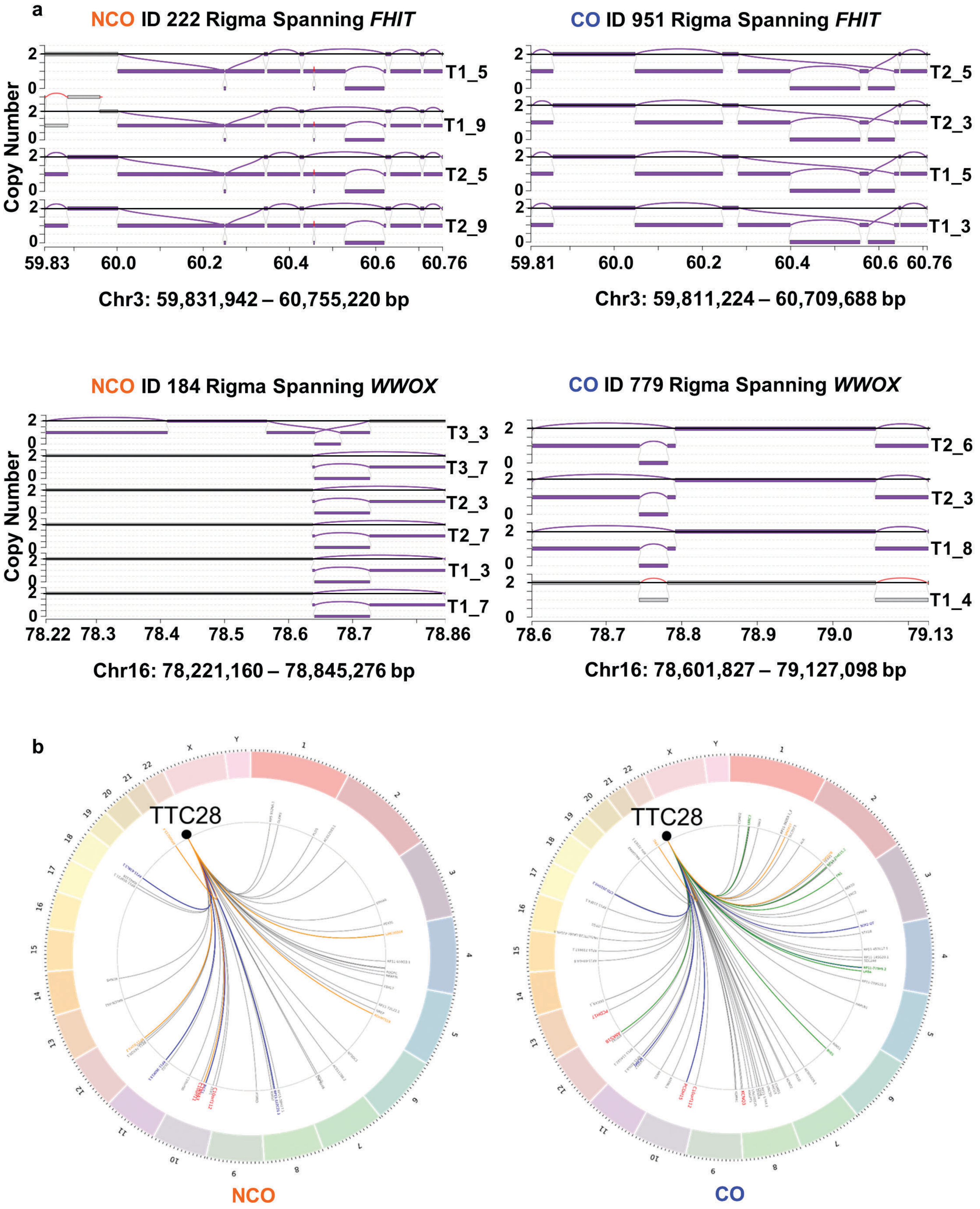

Supplementary Fig. 3. a, Examples of the structural variation feature rigma (Hadi, et al, *Cell*, 2020) spanning regions containing *FHIT* on chromosome 3 and *WWOX* on chromosome 16 in NCO and CO as an early event with clonal SV breakpoints in all biopsies sampled. **b**, Distribution of SV events involving the retrotransposable elements within *TTC28* that disrupted genes throughout the genome. Chromosome numbers are indicated outside the colored ring. Colored links indicate how many samples in the patient with the event had the same fusion: grey (private to a single biopsy), blue (detected in two biopsies), green (detected in three biopsies), orange (detected in all four biopsies). Genes of interest are annotated in red.

Supplementary Fig. 4

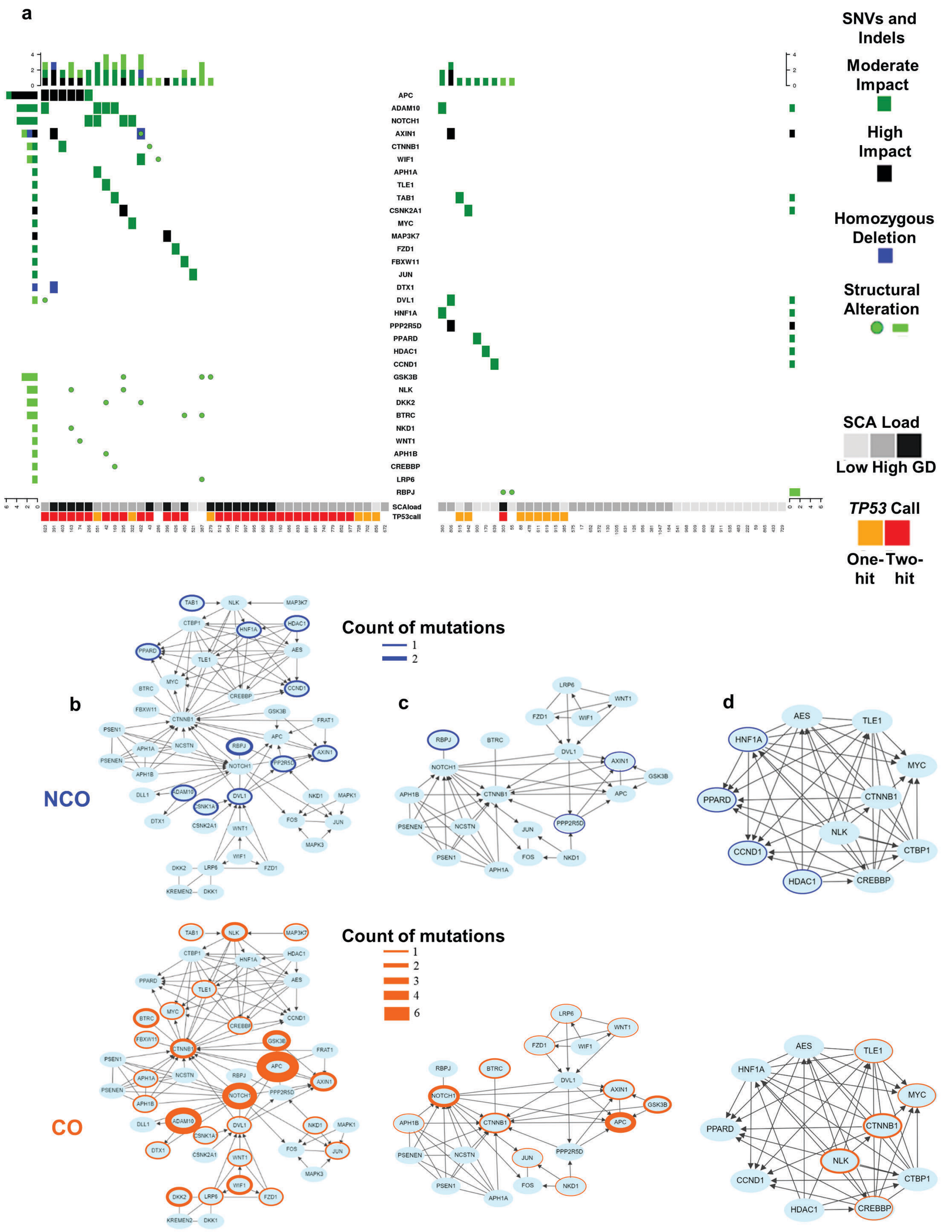

Supplementary Fig. 4. a, Oncoprint of moderate and high impact SNV/Indels, homozygous deletions, and structural variants affecting genes within the “Presenilin action in Notch and WNT signaling pathway” from the Network Data Exchange (NDex) **b,** All nodes and edges connecting genes in the “Presenilin action in Notch and WNT signaling pathway”. Thickness of circled gene names indicates the number of patients with mutations, homozygous deletions, or SVs affecting that gene; orange=CO, blue=NCO. **c,d,** Subnetwork traversing 1-step neighborhoods within the “Presenilin action in Notch and WNT signaling pathway” highlighting differing alterations between CO and NCO. **c.** Subnetwork traversing 1-step neighborhoods from *GSK3B*, *BTRC*, *NKD1*, *WNT1*, *APH1B* and *RBPJ*. These genes were affected only by structural variants within this pathway. **d,** Subnetwork traversing 1-step neighborhoods from *HNF1A*, *PPARD*, *CCND1* and *HDAC1*. Thickness of the oval highlighting gene names corresponds to the number of mutations in each gene.

Supplementary Fig. 5

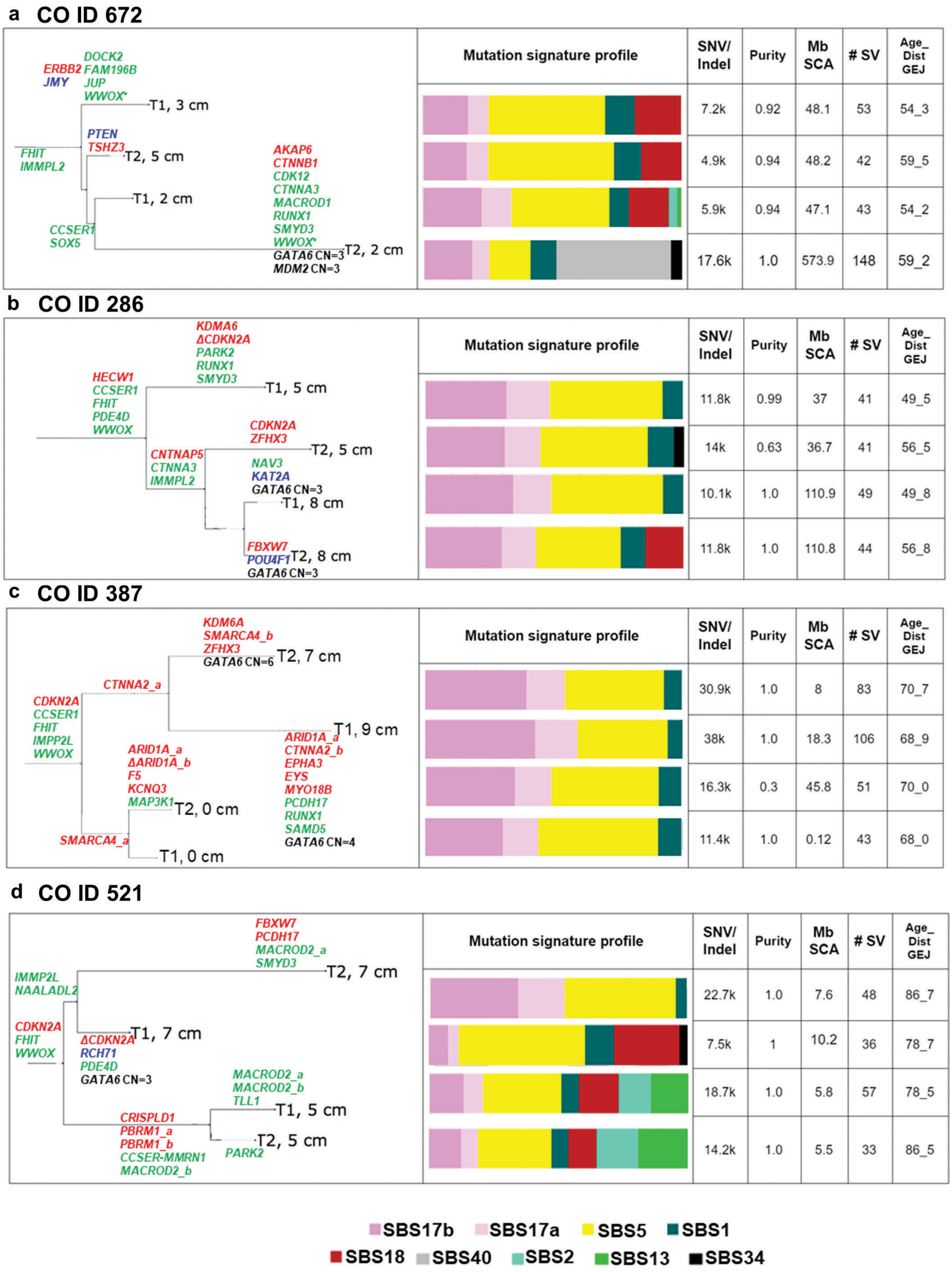

Supplementary Fig. 5. Details from four CO cases (IDs 672, 286, 387, 521) without mutations, deletions or SVs affecting *TP53*. All four had long BE segments (6-16 cm), increasing the potential for missed clones due to a low proportion of the segment having been sampled. Mean and range of CO ages at baseline endoscopy where BE was first diagnosed in our study with and without *TP53* alterations were 64 (32.1-81.1) and 55 (39.4-69.6) years, respectively. In each phylogeny in panels a-d, red genes indicate ESAD "genes of interest" with high or moderate impact SNV or Indel mutations, blue genes have mutations in direct *TP53* effectors, and green genes indicate "ESAD Driver" genes disrupted by structural variants. Chromosome copy number changes on Chr18 spanning *GATA6* are listed in black. ID 521 had a maximum histopathologic diagnosis of low-grade dysplasia at their T1 time point, and IDs 672, 286 and 387 had a maximum diagnosis of indefinite for dysplasia at T1. **a**, ID 672 had low level amplification of *MDM2* that was below the threshold of 9.5 copies that has been proposed for inactivation of *TP53* (Saiki, et al., 2015, *Oncotarget*, v6, pp7701-12); however, the presence of previously detected aneuploid populations, high levels of SVs and somatic chromosome alterations (SCA) suggest *TP53* may be functionally inactivated in this CO case. In addition, previously studied samples from this CO case had several flow-cytometric abnormalities typical of patients with *TP53* mutation. After 13 years of clinical surveillance without flow abnormalities, three small aneuploid populations were detected by flow cytometry five months before cancer diagnosis, two greater than 2.7N and one less than 2.7N; all were minority clones comprising less than 12% of the sorted cells. These ploidies persisted and were detected at the T2 cancer endoscopy. Of note clinically, ID 672 was treated (surgery and radiation) for tonsillar (head and neck, typically HPV) carcinoma, two years prior to their first endoscopy, at which time BE was detected and the patient was referred for upper gastrointestinal endoscopy. ID 672 had a low VAF *PTEN* mutation in one biopsy. **b-d**, Although previously analyzed samples from CO case IDS 286, 387, and 521 had transient, DNA-content flow abnormalities detected either before or at the cancer endoscopy, no biopsies were detected with high levels of SCA characteristic of *TP53* inactivation. The samples analyzed for WGS in this study may represent an alternate pathway to ESAD that does not require *TP53* inactivation, or more likely, sampling error that missed a small *TP53*-altered clone. **b**, CO ID 286 presented with a 16 cm BE segment and was followed six years to cancer diagnosis and surgery. Following surgery, this patient was followed for another nine years to monitor residual BE post esophagectomy. Only one small 2.7N of 3.2% was detected two years before esophagectomy. No DNA-content abnormalities were detected in the 2cm of residual BE. BE persisted after surgery for >14 years until the patient died of unrelated causes. Two biopsies have whole chromosome 18p and q arm copy gain, but the rest of the genome has little SCA. **c**. CO case ID 387 had a history of refluxate into the mouth and duodenal ulcer diagnosis for more than 30 years before BE was diagnosed, suggesting this patient had BE and ulcer for potentially decades before endoscopic surveillance began. After BE diagnosis the CO case was followed for 27 months to cancer with only a transient tetraploidy at baseline endoscopy (T1). BE persisted after surgery for >12 years and the patient lived cancer free for >22 years post surgery. **d,** CO case ID 521 was followed for 16 years with only a single 2.6N of 12.1% at the T2 endoscopy (cancer diagnosis). Cancer was diagnosed as poorly differentiated, signet ring, invasive carcinoma.

Supplementary Fig. 6

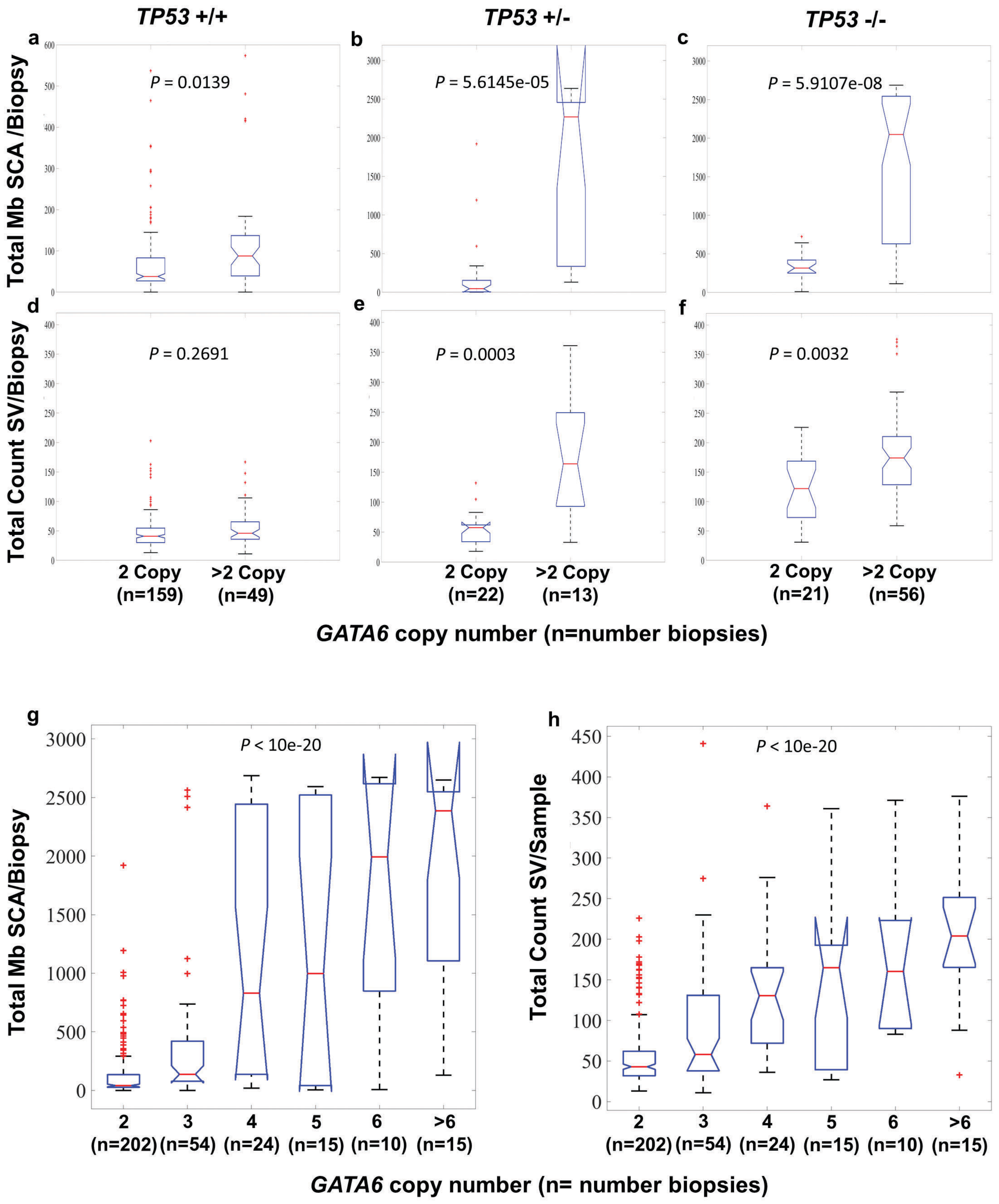

Supplementary Fig. 6. An ESAD risk-associated region on Chr. 18q, including *GATA6*, was identified as an early chromosomal gain event in BE in a previous study (Li et al., *Cancer Prevention Research*, 2014). **a-f,** Biopsies with *GATA6* gain had significantly higher somatic chromosomal alteration (SCA) load, regardless of *TP53* mutation status, and significantly higher structural variant (SV) load with one-hit or two-hit *TP53* alterations, compared to biopsies with no *GATA6* copy gain. Mann–Whitney U test was used for comparison of the two groups. Each dot represents the alteration amount of a biopsy. The number of biopsies per group stratified by TP53 status were represented by n in the plot. Centerline of box plots indicates median, box edges 1st and 3rd quartiles and whiskers 1.5x interquartile range (IQR), all data points outside of IQR were plotted. **g-h**, SCA and SV load increased with increasing number of copies of *GATA6* (Jonckheere-Terpstra trend test, p <10e-20, Box plots settings are same as in a-f). Two-hit *TP53* and *GATA6* gain (>2 copies) were detected together in 17.5% of biopsies, which was a significantly higher frequency than expected (*P* = 2.4x10-5), given the individual frequencies of the two events, suggesting there was positive selection for cell populations in which both two-hit *TP53* alteration and *GATA6* gain occurred. *TP53* alterations were detected in 10.9% (one-hit) and 24.1% (two-hit) of all biopsies, and copy gain spanning the *GATA6* region was detected in 36.9%. However, one-hit *TP53* alterations and *GATA6* gain were detected together in only 4.1% of biopsies, near the expected frequency for these being independent events (dependency test *P* = 0.489). Additionally, detection of biopsies with *TP53* alterations without *GATA6* gain (13.4%) and *GATA6* gain without *TP53* alterations (15.3%) support that one-hit *TP53* and *GATA6* gain events can arise independently from each other. Given the role of *GATA6* in epithelial development and the previous finding of increased risk of future ESAD with early copy gain in the *GATA6* region, the data presented here supports a model in which increasing copies of the chromosomal region containing *GATA6*, in conjunction with *TP53* alterations, promotes progression to ESAD via a genomic instability process resulting in higher SCA and SV load.

Supplementary Fig. 7

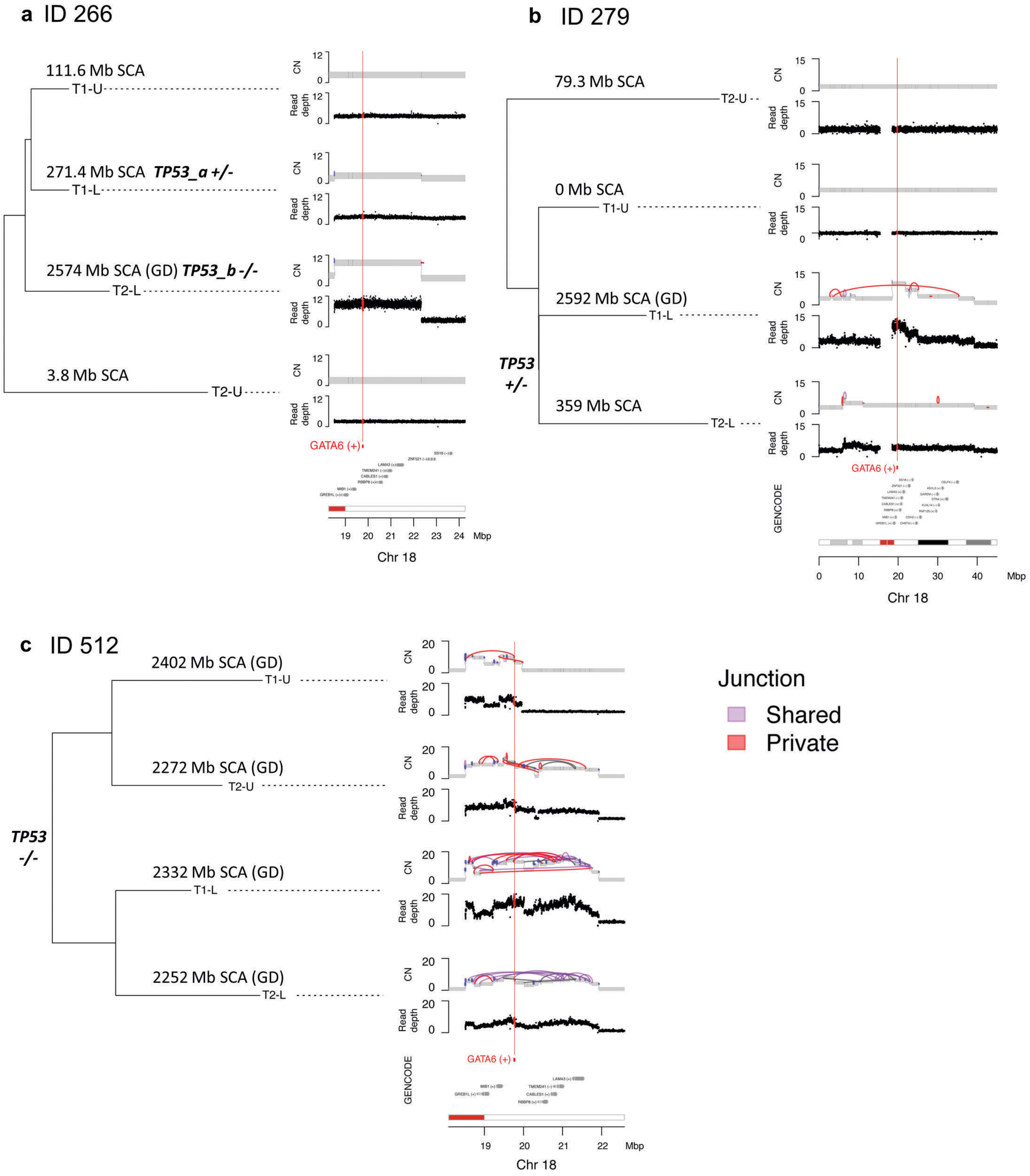

Supplementary Fig. 7. Three patient examples with structural chromosomal alterations within or surrounding the *GATA6* region of chromosome 18 (*GATA6* location, 19.74-19.78 Mbp, indicated by vertical red line extending through tracks). Shown on the right for each panel are parsimony trees based on high confidence somatic SNVs and indels (called by at least two variant callers), with branch lengths reflecting the number of mutations unique to each branch. Tips are annotated for each sample with T1 or T2 (timepoint), and location in the upper (U) or lower (L) third of the Barrett's segment. Additional genes on chromosome 18q:20-40Mb with higher frequency alteration in CO compared to NCO are annotated below the tracks. CN=copy number. Mb SCA= megabases of somatic chromosomal alteration. **a.** ID 266. Two separate *TP53* mutations are indicated by "*TP53* _a" in T1-L (T1 lower) and "*TP53*_b" in T2-L (T2 lower). T1-L has one-hit *TP53* with moderate SCA, whereas T2-L has two-hit *TP53*, genome doubling (GD), and high-level focal gain including *GATA6.* Additional genes are annotated within this region which were found to be altered significantly more frequently in CO compared to NCO including *GREB1L, MIB1, RBBP8, CABLES1, TMEM241, LAMA3, ZNF521, SS18.* **b**. ID 279. T1-L and T2-L samples both share a clonal *TP53* mutation, have moderate SCA or are genome doubled (GD), and show gain in the GATA6 region. Additional genes are annotated within this region which were found to be altered significantly more frequently in CO compared to NCO, including *GREB1L, MIB1, RBBP8, CABLES1, TMEM241, LAMA3, ZNF521, SS18, CHST9, CDH2, RNF125, GAREM, KLHL14, ASXL3, DTNA, CELF4.* **c**. ID 512. A clonal *TP53* mutation was detected at the root (detected in all four samples), each sample has high SCA with genome doubling (GD), and each contains focal, high-level gain around *GATA6.* T1-U and T2-U samples in the upper esophagus are in the top clade, and T1-L and T2-L samples in the lower esophagus are in the bottom clade. Additional genes are annotated within this region which were found to be altered significantly more frequently in CO compared to NCO including *GREB1L, MIB1, RBBP8, CABLES1, TMEM241, LAMA3.*

Supplementary Fig. 8

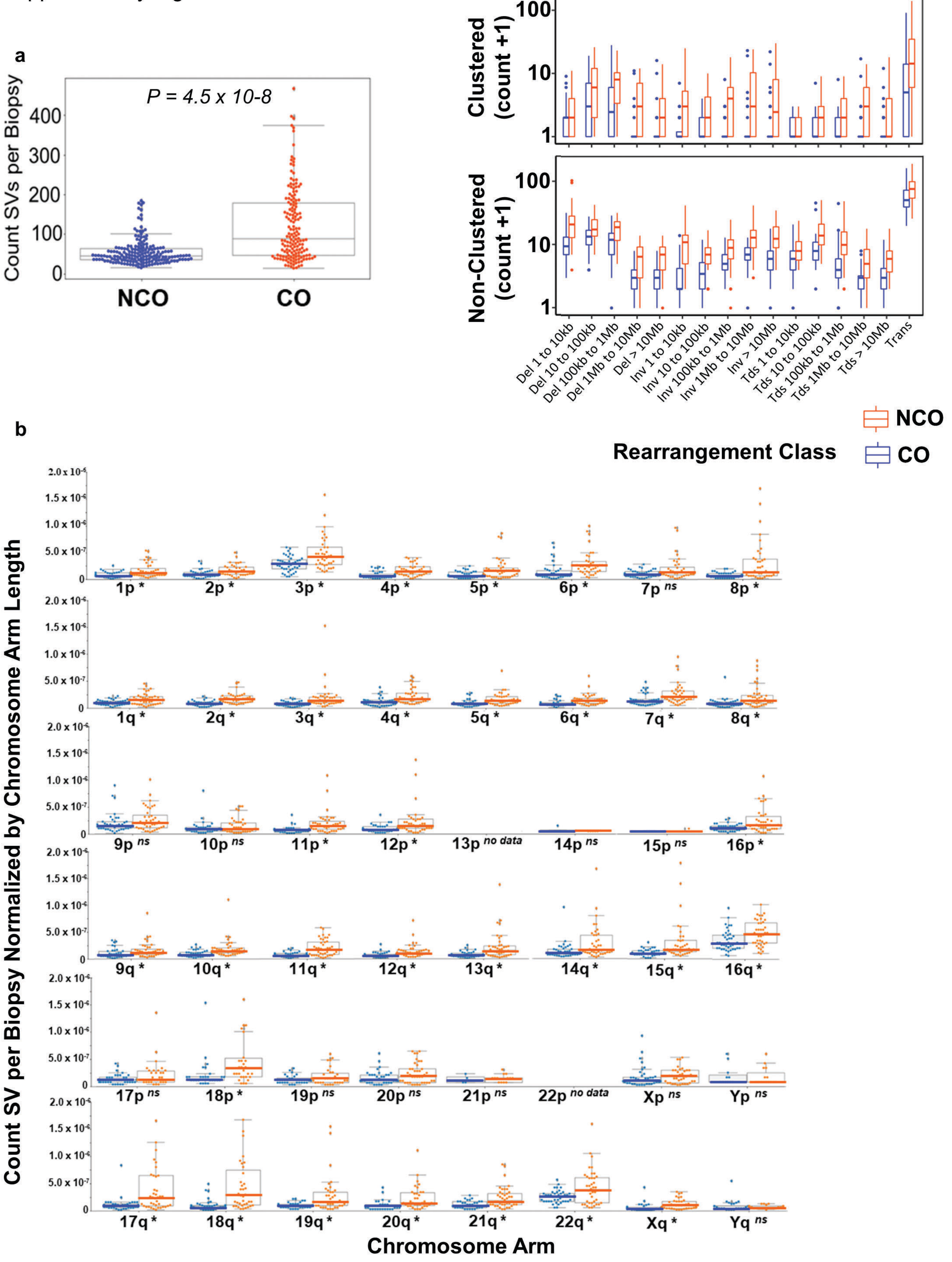

Supplementary Fig. 8 For a-c, each dot represents alteration amount in a biopsy. Box centerline of box plots indicates median, box edges 1st and 3rd quartiles and whiskers 1.5x interquartile range (IQR), all data points outside of IQR were plotted. **a,** Count of SVs per biopsy in NCO and CO. Mann–Whitney U test was used for comparison of the two groups. **b,** SV count per sample on each chromosome arm, normalized by chromosome arm length. **c,** Count of SV in NCO and CO in 32 rearrangement classes based on SV type, size and clustering of rearrangements (Nik-Zainal, et al. *Nature*, 2016) in NCO and CO. Patients with CO had significantly higher levels of both clustered SVs (combined classes, P = 0.00015) and all nonclustered SV categories (analyzed individually, all P<0.0001), indicating the increased SV counts occur across all SV categories. Notably, clustered and nonclustered translocations (often involving the TTC28 locus (Pitkänen et al. *Oncotarget*, 2014)) and small nonclustered deletions were frequent SV classes in both NCO and CO. Del, deletion; Inv, inversion; Tds, tandem duplications.

Supplementary Fig. 9

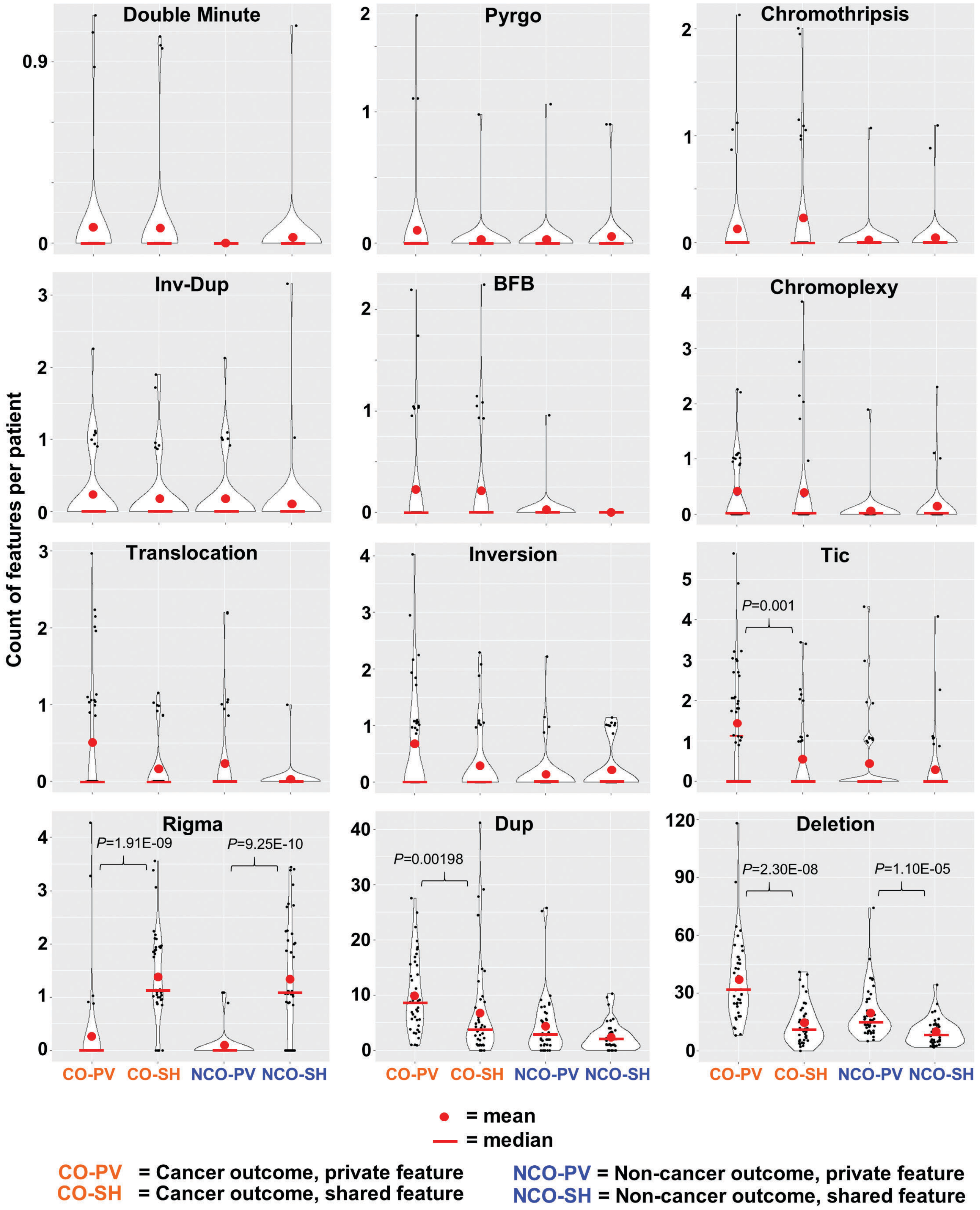

Supplementary Fig. 9. Within either CO or NCO, median load of each structural variant feature per patient was compared for features that were private (PV) to a single biopsy, or shared (SH) between two to four biopsies in that patient. FDR 0.01. Tyfonas is not shown due to low event frequency. All private vs shared comparisons without a *P*-value were non-significant at $P < 0.01$ level.

Supplementary Fig. 10

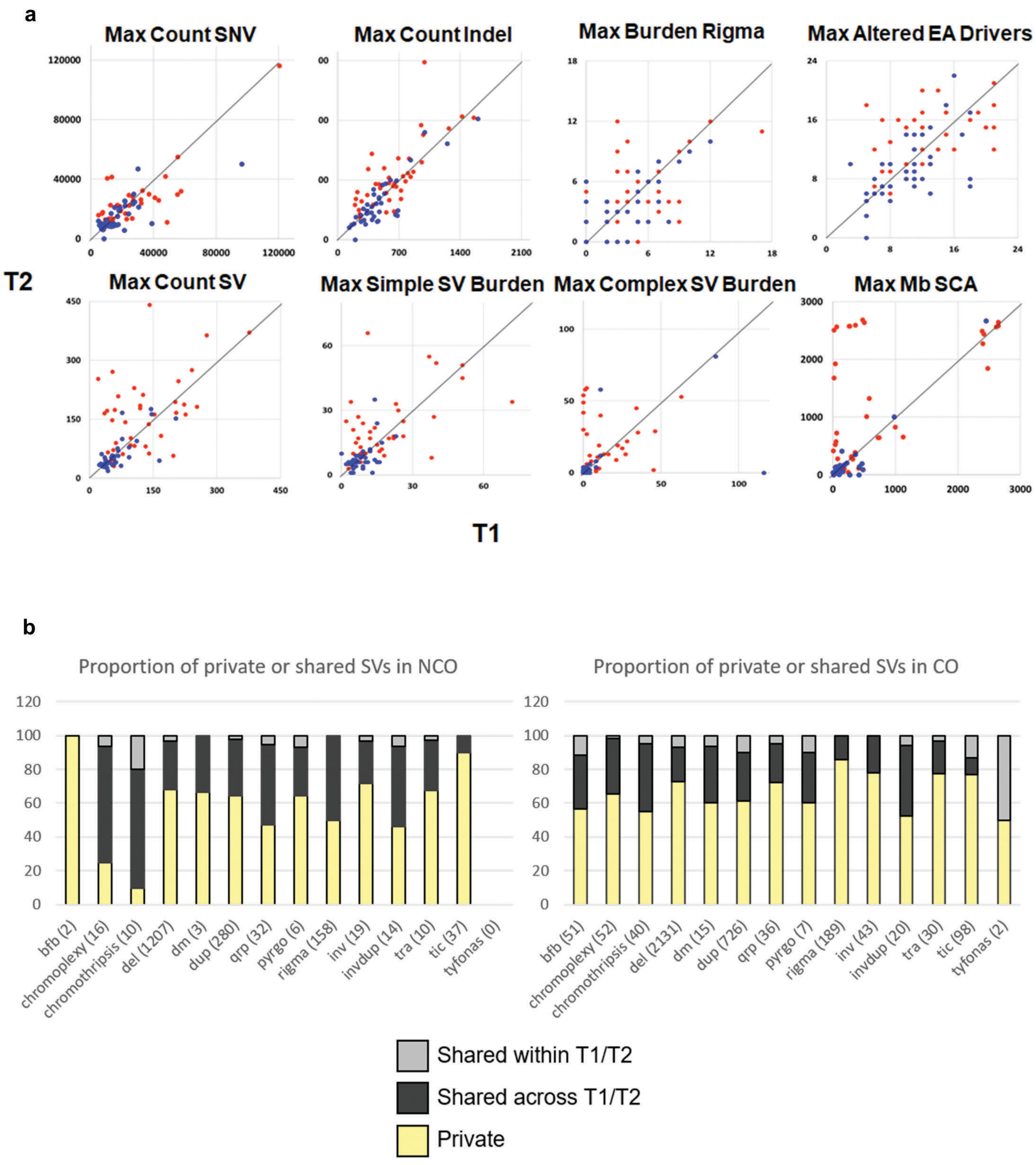

Supplementary Fig. 10. Distribution of genome alterations across time and space. A) Each plot point represents data from one patient (blue dots represent NCO, orange dots CO) where the value on the x-axis represents the maximum measure from the T1 biopsies and the value on the y-axis represents the maximum measure from the T2 biopsies. Diagonal line indicates equal values for T1/T2. Points above the diagonal indicate patients where the max T2 biopsy had more events than the max T1 biopsy. B) Proportion of private vs shared SVs by SV type in NCO and CO patients. Numbers after column names indicate the total number of events observed for each SV type. Shared within T1/T2 indicates the same SV was observed in both biopsies from either T1 or T2; shared across T1/T2 indicates the same SV was observed in one or more biopsies from both T1 and T2; Private indicates SV was only observed in a single biopsy.

Supplementary Methods

Sample Processing

2.5M Illumina SNP array

Somatic chromosome alterations (chromosome copy number changes and cnLOH) were assessed in BE and normal biopsies using the Omni 2.5M 8v1.3 array (Illumina) following manufacturer recommendations. One sample (391-23521-155R-31) was evaluated using Omni 2.5M-8 v1.2. For copy number, B-allele frequency at heterozygous loci, and allele-specific copy number, BE biopsies were paired to their patient's normal control sample in Genome Studio with Partek Plug-in v2.13.1[1].

WGS library preparation and direct sequencing

For 78 patients and 417 biopsies, whole genome sequencing (WGS) libraries were prepared using the TruseqDNA PCR-free Library Preparation Kit (Illumina) in accordance with the manufacturer's instructions. Briefly, 1ug of DNA was sheared using a Covaris LE220 sonicator (Adaptive Focused Acoustics). DNA fragments underwent bead-based size selection and were subsequently end-repaired, adenylated, and ligated to Illumina sequencing adapters. Final libraries were evaluated using fluorescent-based assays including qPCR with the Universal KAPA Library Quantification Kit and Fragment Analyzer (Advanced Analytics) or BioAnalyzer (Agilent 2100). Libraries were sequenced on an Illumina HiSeqX sequencer at 2 x 150bp cycles using v2.5 chemistry.

TruSeqNano library preparation and sequencing

In two patients (ID 322 and ID 360), one or more biopsies had insufficient DNA to perform PCR-free library preparation and no replacements available. Therefore, WGS libraries were prepared using the Illumina TruSeq Nano DNA Library Preparation Kit in all biopsies in these two patients (N=8) and their two normal control samples, in accordance with the manufacturer's instructions. Briefly, 100ng of DNA was sheared using the Covaris LE220 sonicator (Adaptive Focused Acoustics). DNA fragments underwent end-repair, bead-based size selection, adenylation, and Illumina sequencing adapter ligation. Ligated DNA libraries were enriched with PCR amplification (using 8 cycles). Final libraries were evaluated and sequenced as above.

Sample QC Preprocessing

BE and matched normal DNA sequencing data were preprocessed using the Broad "best practices" pipeline, which includes aligning reads to the GRCh37 human reference genome using the BurrowsWheeler Aligner (BWA) [2], marking of duplicate reads by the use of Picard tools (http://picard.sourceforge.net); realignment around indels (done jointly for all biopsies derived from one individual, e.g. four or six BE biopsies, gastric test sample, and matched blood or control gastric normal sample) and base recalibration via Genome Analysis Toolkit (GATK) [3].

DNA sequencing metrics

A battery of Picard (QualityScoreDistribution, MeanQualityByCycle, CollectBaseDistributionByCycle, CollectAlignmentSummaryMetrics, CollectInsertSizeMetrics, CollectGcBiasMetrics) and GATK (FlagStat,

ErrorRatePerCycle) metrics were run on all samples. Custom R scripts and bedToolsCoverage were used to compute sequencing depth of coverage. Outlier detection was performed to identify biopsies requiring manual review. All biopsies included in this study passed these QC metrics.

Sample contamination and normal concordance

All samples used in this study were verified to not be contaminated using VerifyBamId [4], 347 BE samples had low evidence for contamination (median 0.2% (range 0-0.5%), and all 80 normals had low evidence for contamination (median 0.2% (range 0-0.7%, Supplementary Data File 33). To avoid incorrect pairing of BE biopsies and normal control samples, a small panel of polymorphic SNP markers were genotyped in all BE, test gastric and normal control blood/gastric samples and concordance was computed for all pairs. Polymorphic SNVs were taken from release 3 of the 1000 Genome Project [5], and defined as having MAF >= 40%, being within Agilent SureSelect exome targets, and with pairwise LD > 0.8. This allowed detection of gross sample mismatches. All samples included in this study had a minimum concordance of 99.8% with their paired normal control sample (Supplementary Data File 34).

Blood vs. gastric normal control

In addition to the blood normal control, a normal gastric sample from the fundus (upper stomach) in seven individuals was sequenced at 60X in the same experiments as that patient's BE biopsies and treated as if it were a BE biopsy (i.e. paired with the blood normal). In these seven gastric samples only 27 - 43 (mean 34) somatic mutations per

patient were detected, with only 0-2 mutations shared between the gastric sample and all BE biopsies, well within the range of noise. To assess how likely it would be to have five random biopsies share a mutation, a random sampling of five BE samples were compared to check the frequency of the same SNV/Indel mutation appearing in all five samples. Out of 200,000 runs, a maximum of only three mutations were shared in all five randomly selected biopsies. Thus, in the patients for whom only gastric biopsies were available as the normal control, it was concluded random mutations in the normal gastric would have no significant effect on the mutational profile of their paired BE samples.

Sample purity and ploidy

Purity and ploidy for each sample were determined by a modified version of ASCAT [6], with a gamma of 500. Accuracy of the calls of copy number change was determined by comparison to a custom algorithm [1], which provides 1 Mb resolution for copy number changes but does not give an overall purity and ploidy. In cases where the presence of multiple cell populations reduced the accuracy of the ASCAT calls, manual QC was performed that took into account the likelihood of aneuploidy cell populations (based upon the overall DNA content flow cytometric data from that patient, data not shown) as well as evaluation of allele specific copy number differences and relatedness to other samples from the same patient with less ambiguity in their purity/ploidy determinations from the same patient.

Since ASCAT determines purity based upon copy number alterations and cnLOH, it is not able to accurately score purity if a sample contains a large percentage of cells

derived from non-Barrett's epithelium, e.g., mixed with gastric cardia if the sample was near the GEJ. If a sample displayed evidence of non-BE cell contamination, such as a VAF distribution with a sharp peak around 0.1 rather than around 0.5, and also had a low mutation load (2+ caller mutation load in lowest decile (<5,296 mutations)), purity values derived from mutation based algorithms (NYGC) were used for those samples.

SNV and INDEL calling details

Somatic SNVs were called by muTect v1.1.7 [7], Strelka v1.0.14 [8] and LoFreq v2.1.3a [9]. Indels were called using Strelka, and somatic versions of Pindel v0.2.5 [10] and Scalpel v0.5.3 [11]. For all analyses only SNVs and indels that were called by at least two callers (2+) were considered in this study (Supplementary Data File 36). This conservative approach will under-call a proportion of low coverage and low VAF mutations made by single callers. SNVs and indels were filtered using the default filtering criteria of each of the callers. For Pindel and Scalpel (natively germline callers) custom in-house scripts were used for filtering. For all callers except muTect, we required FILTER=PASS; for muTect we required PASS in the filter field of the VCF file. Triallelic positions were removed as some of the SNV callers (e.g. muTect) remove them by default, and previous experience has shown that a high proportion of triallelic sites are due to technical errors. In addition, a subset of artifactual calls were removed by the use of a blacklist created by calling somatic variants on 16 random pairings of 80x/40x in-house sequenced HapMap WGS data.

Common germline variants

The resulting set of SNVs and indels was further filtered to remove common variants seen at MAF ≥ 5% in *DNMT3A, TET2, JAK2, ASXL1, TP53, GNAS, PPM1D, BCORL1* and *SF3B1* genes (see Xie et al., 2014 [12]) or at MAF ≥ 1% elsewhere in the genome, as reported in the 1000 Genomes Project release 3 [5] and the Exome Aggregation Consortium (ExAC) server (http://exac.broadinstitute.org), because these are considered unlikely to be important in cancer.

Unified Allele Count filter

Because callers often return different ref/alt allele counts for the same variant, a unified allele count (UAC) was used. Computation of UAC was based on the bamreadcount tool [13]. For each variant, four values were generated that were independent of callers: tumorref, tumoralt, normalref, normalalt. If the tumor_VAF < normal_VAF the variant was discarded.

SNV and Indel annotation

Mutations were annotated by SnpEff version 4.2 (build 2015-12-05) to associate each mutation with Ensembl 75 transcripts, as well as the predicted impact (High, Moderate, Low, or Modifier) these mutations would have on these transcripts. "Functional" mutations were defined as High or Moderate impact. When multiple annotations were given for the same mutation, the most severe was used.

Unless indicated otherwise, "mutations" are defined as SNVs and indels, and "unique mutations" as the collection of all mutations within a patient, removing duplicates.

Mutations were classified as private if found in only one biopsy of a given patient and shared if they were found in more than one. Mutations were deemed identical if they shared chromosome, position, and REF to ALT change.

Gene name aliases

Gene names used by somatic SNV and indel callers, structural variant callers, and pathway sources were brought in concordance with one another. HUGO gene names were used as the standard and a list of aliases for each gene was compiled. Gene names from pathways were cross referenced with the Hugo gene names; those names that didn't match were further investigated using GenCode (v18) and UniProt. Any remaining gene names manually curated to either convert them to our target list or dropped as not being genes. The final target list included 6,293 gene names across the 321 pathways. Pathway names are listed in Supplementary Data File 16.

Telomere length estimation

To determine average telomere length in each extracted epithelial isolated DNA, a novel method (Telomeasure) was developed that uses chromosome arm-level coverage to infer the number of telomeres and alignment to mock-human telomeres to estimate telomeric DNA content (https://github.com/nygenome/telomeasure) (https://zenodo.org/badge/latestdoi/297955889). Reads with two consecutive telomeric repeats - TTAGGG - were aligned with the fast and exhaustive GEM mapper [14]. Because GEM can return gapped alignments, non-canonical telomeric repeats can be detected. Mapping position of the telomeric read is also reported for use in identifying

genomic rearrangements that relocate telomeres. Telomeasure estimated average telomere length. Initially, duplicate reads are filtered and coverage_chromosome arm/coverage_autosomes and GC-matched coverage_autosomes is estimated. Coverage_ autosome is compared to coverage_chromosome arm to estimate telomere count (Equation 1). Putative telomeric sequence in WGS read is quantified as telo-aligned length. Finally, average telo-length is estimated (Equation 2).

Equation 1

$$telomere = \sum_{i=1}^{46} \frac{coverage_{chromarm(i)}}{coverage_{autosomes}}$$

Equation 2

$$average\ telo - length = \frac{telo - aligned\ length}{telomere_{count} x\ GC\ matched\ coverage_{autosomes}}$$

Mutation Signature Analysis Solutions

SigProfiler was utilized to identify the number of operative mutational signatures across the examined BE samples (Supplementary Data File 35). The tool identified solutions with 1, 2, 3, 4, 5, 6, 8 and 9 operative signatures as stable and reproducible (minimum stability >0.80 [15]). Statistically, the solution with 9 signatures did not provide a better description of the data compared to the solution with 8 signatures (p-value: 0.0976) but it did describe the data better than using 7 signatures (p-value: 3.77E-19). Statistical comparisons were performed using Mann–Whitney U tests comparing the distributions of the cosine similarities between the patterns of samples reconstructed with 7 and 8 signatures, respectively, with the patterns of the originally examined samples. Cosine

similarities were calculated based on SBS-96 classification while taking into account the sample patterns [16]. SigProfiler selected 8 signatures as the optimal solution since this is a stable solution which describes the data just as well as the stable solution with maximum number of signatures.

Simple SV calling overview (NYGC)

SVs were called with Crest v1.0 [17], Delly v0.6.1 [18], and BreakDancer v1.4.0 [19]. Known germline SVs were filtered out, with corroborating breakpoints used to generate a “high confidence” list of SVs. Only these high confidence SVs were considered in this study (Supplementary Data File 36).

Filtering and annotation of SVs

All filtering and annotation of SVs was performed based on bedtools (http://bedtools.readthedocs.org). SVs called by Crest, Delly, and BreakDancer were merged and annotated using BEDPE format. Two SV calls were merged if they shared at least 50% reciprocal overlap (for intrachromosomal SVs only), their predicted breakpoints were within 300bp of each other and breakpoint strand orientation matched for both breakpoints. Thus, merging was done independent of which SV type was assigned by the SV caller. After merging, each SV was annotated with the closest CNV changepoint as detected by NBICseq [20] from read depth signals. This added confidence to true SV breakpoints that were not copy neutral. Additionally, an independent sensitive split read check was applied to each breakpoint using SplazerS [21]. Apart from adding confidence and base-pair precision to the breakpoint, this step increased removal of

remaining germline SVs also found in the normal control. To minimize the high rate of false positive SV calls due to undetected germline SVs or systematic artifacts due to mapping ambiguities, germline SVs were annotated and filtered through overlap with known SVs [5] as well as through overlap with an in-house blacklist of SVs derived from germline SVs and artifacts called in healthy genomes. Finally, SVs were prioritized if they were called by more than one tool or called by only one tool but also confirmed by 1) a CNV changepoint, or 2) at least three split reads (in BE samples only). In addition, all high-confidence Crest calls were retained due to the specificity of Crest-only high-confidence calls. All predicted copy number and SVs were annotated with gene overlap (RefSeq, Cancer Census) and potential effect on gene structure (e.g. disruptive, intronic, intergenic). If a predicted SV was found to disrupt two genes, and strand orientations were compatible, the SV was annotated as a putative gene fusion candidate. Reading frame was not considered. Further annotations included sequence features within breakpoint flanking regions, e.g. mappability, simple repeat content, segmental duplications and Alu repeats.

For the purpose of pathway analysis, determination of disruption of a gene by an SV event was performed using NYGC pipeline calls “disrupt L”, “disrupt R”, or “fusion” to indicate disruption of the coding sequence by a structural variation. Similarly, a finding of HD that disrupted one or more exons in a gene was considered to be a functional disruption of the gene and the gene was considered to be mutated.

Classes of SVs

Classifying SVs into features to fit to known rearrangement signatures was performed as described [22]. Briefly, SV classes for each rearrangement was determined for the following features: junction clustering, type, and size. Clustered rearrangements were identified by candidate junction groups with average inter-breakpoint distances (determined via piecewise constant fitting, with hyperparameters set to gamma = 25, and k_min = 10) that were 10x less than the genome-wide average distance. Junction types were categorized as tandem duplications, deletions, inversion or translocations, depending on the orientation and chromosomal location of breakpoints. Junction sizes for those rearrangement types which are intrachromosomal were measured in the ranges 1-10 kbp, 10-100 kbp, 100 kbp - 1 Mbp, 1Mbp - 10 Mbp, and > 10 Mbp. Upon calculating the per sample SV catalogs based on these (32 total) features, SV signatures were determined by fitting the six known SV signature weights [22] to the rearrangement counts via non-negative least squares in R (package nnls).

Calling complex chromosomal events

Chromosomal complex events were annotated as described in Hadi, et al. 2020 [23]. Briefly, Junction Balance Analysis (JaBbA) was performed on 347 samples across the cohort with merged structural variants called by SvAbA [24], Crest, Breakdancer, and DELLY, and the ratio of tumor to normal GC- and mappability-corrected read depths calculated across 200 bp bins. SvAbA junctions were co-called across samples jointly per patient. For SvAbA, only those junctions with both the FILTER field marked as PASS and with at least 5 supporting reads in any sample were used. For all junctions,

those across samples overlapping within 300 base pairs that had identical breakend orientations were considered to be present in the samples found.

Junction-balanced genome graphs consist of estimates of both interval and junction copy number resolved genome-wide as well as loose ends, which are coverage changes unexplained by a structural variant. Heuristics to annotate simple and complex events were applied to genome graphs to annotate the following: simple deletions, duplications, translocations, inverted duplications. Complex: templated insertion chains (TIC), chromoplexy, chromothripsis, breakage fusion bridge cycles, double minutes, and tyfonas. Heuristics are described in Hadi et al 2020 [23], and are available in the gGnome R package at https://github.com/mskilab/gGnome.

For track data, GC- and mappability-corrected read depth data were collapsed into 1 kbp bins using the median and processed through Dryclean [25] (https://github.com/mskilab/dryclean) to isolate the coverage changes attributed to somatic CNV, and to de-noise the coverage profiles. JaBbA models per sample were re-fit jointly across samples using the "balance" function in gGnome within the subgraphs.

Anomalous biopsies

Nine of 340 biopsies, (four CO and five NCO biopsies, two of which were from the T3 time point) were in the lowest 5% of mutation load (<4,285 mutations), and lacked hallmarks of BE: no observed chromosome 9p cnLOH or loss, no high-confidence SVs, deletions, or loss in *TP53, FHIT, CDKN2A*, or *WWOX*, and no *TP53* alterations of any

kind (Supplementary Data File 5). We hypothesize that these "atypical" biopsies may have little to no Barrett's epithelium, but in the absence of corroboration have included them in all analyses unless otherwise indicated.

QUANTIFICATION AND STATISTICAL ANALYSIS EXTENDED METHODS

Phylogenetic Analysis

We constructed maximum parsimony trees of the four biopsies per patient based on SNVs only, using *dnapars* from Phylip v3.695 [26]. Each biopsy was classified as 'distal' or 'proximal' based on the relative positions of the four biopsies: biopsies closer to the most distal biopsy than to the most proximal were classified as "distal", and those closer to the most proximal biopsy were classified as "proximal." Any biopsy equidistant between the two was placed in the group with fewer biopsies. This resulted in 55/80 patients with two distal biopsies and two proximal biopsies, 24 patients with three of one type and one of the other, and one patient where all four biopsies were equidistant from the GEJ. For spatial phylogenetic analysis we used only the 55 patients with two distal and two proximal biopsies, while for temporal analysis we used all 80 patients. We computed expected numbers of each of the six possible arrangements of two pairs of biopsies (T1/T2 for temporal analysis, proximal/distal for spatial analysis) assuming random branching-process phylogenies with random assignment of tip labels, and compared the observed values to this random expectation using a chi-squared test. In

addition, we used a chi-square test to determine whether the CO patient topologies differed significantly from the NCO topologies in either spatial or temporal structure.

Support for the trees was determined based on 1000 bootstrap replicates made using *seqboot* and *consense* from Phylip v3.695 [27]. Bootstrap support was generally high. In 65/80 patients all branches had 100% bootstrap support, with 73/80 patients with bootstrap support above 850/1000 for all nodes, which has been suggested as a reasonable approximation of a 95% confidence interval. No patient showed more than 15% support for a different T1/T2 topology class. One patient (of the 55 with two distal and two proximal biopsies) showed more than 15% support for a different proximal/distal topology class. Removing that patient from the analysis did not change the conclusion, nor did removing all 15 patients with <100% support for any node (data not shown).

Literature cited in Extended Methods

1. Li, X. *et al.* Temporal and spatial evolution of somatic chromosomal alterations: a case-cohort study of Barrett's esophagus. *Cancer Prev. Res.* **7**, 114–127 (2014).
2. Li, H. & Durbin, R. Fast and accurate short read alignment with Burrows-Wheeler transform. *Bioinformatics* **25**, 1754–1760 (2009).
3. McKenna, A. *et al.* The Genome Analysis Toolkit: a MapReduce framework for analyzing next-generation DNA sequencing data. *Genome Res.* **20**, 1297–1303 (2010).
4. Jun, G. *et al.* Detecting and estimating contamination of human DNA samples in

sequencing and array-based genotype data. *Am. J. Hum. Genet.* **91**, 839–848 (2012).

5. Genomes Project, Consortium *et al.* An integrated map of genetic variation from 1,092 human genomes. *Nature* **491**, 56–65 (2012).
6. Smith, L. P., Yamato, J. A. & Kuhner, M. K. CNValidator: validating somatic copy-number inference. *Bioinformatics* **35**, 2660–2662 (2019).
7. Cibulskis, K. *et al.* Sensitive detection of somatic point mutations in impure and heterogeneous cancer samples. *Nat. Biotechnol.* **31**, 213–219 (2013).
8. Saunders, C. T. *et al.* Strelka: accurate somatic small-variant calling from sequenced tumor-normal sample pairs. *Bioinformatics* **28**, 1811–1817 (2012).
9. Wilm, A. *et al.* LoFreq: a sequence-quality aware, ultra-sensitive variant caller for uncovering cell-population heterogeneity from high-throughput sequencing datasets. *Nucleic Acids Res.* **40**, 11189–11201 (2012).
10. Ye, K., Schulz, M. H., Long, Q., Apweiler, R. & Ning, Z. Pindel: a pattern growth approach to detect break points of large deletions and medium sized insertions from paired-end short reads. *Bioinformatics* **25**, 2865–2871 (2009).
11. Narzisi, G. *et al.* Accurate de novo and transmitted indel detection in exome-capture data using microassembly. *Nat. Methods* **11**, 1033–1036 (2014).
12. Xie, M. *et al.* Age-related mutations associated with clonal hematopoietic expansion and malignancies. *Nat. Med.* **20**, 1472–1478 (2014).
13. Larson, D. E. *et al.* SomaticSniper: identification of somatic point mutations in whole genome sequencing data. *Bioinformatics* **28**, 311–317 (2012).
14. Marco-Sola, S., Sammeth, M., Guigó, R. & Ribeca, P. The GEM mapper: fast,

accurate and versatile alignment by filtration. *Nat. Methods* **9**, 1185–1188 (2012).

15. Alexandrov, L. B. *et al.* The repertoire of mutational signatures in human cancer. *Nature* **578**, 94–101 (2020).
16. Bergstrom, E. N., Barnes, M., Martincorena, I. & Alexandrov, L. B. Generating realistic null hypothesis of cancer mutational landscapes using SigProfilerSimulator. *BMC Bioinformatics* **21**, 438 (2020).
17. Wang, J. *et al.* CREST maps somatic structural variation in cancer genomes with base-pair resolution. *Nat. Methods* **8**, 652–654 (2011).
18. Rausch, T. *et al.* DELLY: structural variant discovery by integrated paired-end and split-read analysis. *Bioinformatics* **28**, i333–i339 (2012).
19. Fan, X., Abbott, T. E., Larson, D. & Chen, K. BreakDancer: Identification of Genomic Structural Variation from Paired-End Read Mapping. *Curr. Protoc. Bioinformatics* **45**, 15.6.1–11 (2014).
20. Xi, R., Lee, S., Xia, Y., Kim, T.-M. & Park, P. J. Copy number analysis of whole-genome data using BIC-seq2 and its application to detection of cancer susceptibility variants. *Nucleic Acids Res.* **44**, 6274–6286 (2016).
21. Emde, A.-K. *et al.* Detecting genomic indel variants with exact breakpoints in single- and paired-end sequencing data using SplazerS. *Bioinformatics* **28**, 619–627 (2012).
22. Nik-Zainal, S. *et al.* Landscape of somatic mutations in 560 breast cancer whole-genome sequences. *Nature* **534**, 47–54 (2016).
23. Hadi, K. *et al.* Distinct Classes of Complex Structural Variation Uncovered across Thousands of Cancer Genome Graphs. *Cell* **183**, 197–210.e32 (2020).

24. Wala, J. A. *et al.* SvABA: genome-wide detection of structural variants and indels by local assembly. *Genome Res.* **28**, 581–591 (2018).

25. Deshpande, A., Walradt, T., Hu, Y., Koren, A. & Imielinski, M. Robust foreground detection in somatic copy number data. *bioRxiv* (2019).

26. Felsenstein, J. *PHYLIP (Phylogeny Inference Package)*. (Distributed by the author. Department of Genome Sciences, University of Washington, 2005).

27. Felsenstein, J. Confidence limits on phylogenies: an approach using the bootstrap. *Evol. Appl.* **39**, 783–791 (1985).
