# Supplementary material for: Somatic whole genome dynamics of precancer in Barrett’s esophagus reveals features associated with disease progression
Source: Nat Commun. 2022 Apr 28;13:2300. doi: 10.1038/s41467-022-29767-7 (PMC9050715; doi:10.1038/s41467-022-29767-7)
Supplement: Supplementary file 7 — Reporting Summary [file 41467_2022_29767_MOESM7_ESM.pdf]

nature portfolio

Corresponding author(s): Thomas Paulson, Xiaohong Li

Last updated by author(s): Mar 18, 2022

Reporting Summary

Nature Portfolio wishes to improve the reproducibility of the work that we publish. This form provides structure for consistency and transparency in reporting. For further information on Nature Portfolio policies, see our Editorial Policies and the Editorial Policy Checklist.

Statistics

For all statistical analyses, confirm that the following items are present in the figure legend, table legend, main text, or Methods section.

n/a	Confirmed	
☐	☒	The exact sample size (*n*) for each experimental group/condition, given as a discrete number and unit of measurement
☐	☒	A statement on whether measurements were taken from distinct samples or whether the same sample was measured repeatedly
☐	☒	The statistical test(s) used AND whether they are one- or two-sided<br>*Only common tests should be described solely by name; describe more complex techniques in the Methods section.*
☐	☒	A description of all covariates tested
☐	☒	A description of any assumptions or corrections, such as tests of normality and adjustment for multiple comparisons
☐	☒	A full description of the statistical parameters including central tendency (e.g. means) or other basic estimates (e.g. regression coefficient) AND variation (e.g. standard deviation) or associated estimates of uncertainty (e.g. confidence intervals)
☐	☒	For null hypothesis testing, the test statistic (e.g. *F*, *t*, *r*) with confidence intervals, effect sizes, degrees of freedom and *P* value noted<br>*Give P values as exact values whenever suitable.*
☒	☐	For Bayesian analysis, information on the choice of priors and Markov chain Monte Carlo settings
☒	☐	For hierarchical and complex designs, identification of the appropriate level for tests and full reporting of outcomes
☒	☐	Estimates of effect sizes (e.g. Cohen's *d*, Pearson's *r*), indicating how they were calculated

Our web collection on statistics for biologists contains articles on many of the points above.

Software and code

Policy information about availability of computer code

Data collection	BE and matched normal DNA sequencing data were preprocessed using the Broad “best practices” pipeline, which includes aligning reads to the GRCh37 human reference genome using the BurrowsWheeler Aligner (BWA), marking of duplicate reads by the use of Picard tools (http://picard.sourceforge.net); realignment around indels (done jointly for all biopsies derived from one individual, e.g. four or six BE biopsies, gastric test sample, and matched blood or control gastric normal sample) and base recalibration via Genome Analysis Toolkit (GATK).
Data analysis	bamreadcount; Larson 2012; https://bioweb.pasteur.fr/packages/pack@bam-readcount@0.4.6<br>bedToolsCoverage v2.26.0; Quinlan and Kindlon; https://bedtools.readthedocs.io/en/latest/content/tools/coverage.html<br>"BreakDancer v1.4.0NYGC pipeline version 4 (February 12, 2016); Chen et al. 2009; https://github.com/genome/breakdancer/releases/tag/v1.4.0"<br>BurrowsWheeler Aligner (BWA)aln; Li and Durbin, 2009; http://bio-bwa.sourceforge.net/<br>CHASM; Carter et al. 2009; http://wiki.chasmsoftware.org/index.php/Main_Page<br>CNValidator v1.0; Smith et al. 2018; https://github.com/kuhnerlab/CNValidator<br>copynumber v1.17.0; Smith et al. 2018; https://github.com/kuhnerlab/copynumber<br>"Crest v1.0NYGC pipeline version 4 (February 12, 2016); Wang et al. 2011; https://www.ncbi.nlm.nih.gov/projects/gap/cgi-bin/study.cgi?study_id=phs000340.v1.p1"<br>deconstructSigs; Rosenthal et al. 2016; https://github.com/raerose01/deconstructSigs<br>"Delly v0.6.1NYGC pipeline version 4 (February 12, 2016); Rausch et al. 2012; https://tobiasrausch.com/delly/"<br>dN/dScv v0.0.1.0; Martincorena I, et al. (2017; http://www.cell.com/cell/fulltext/S0092-8674(17)31136-4)<br>Dryclean; Deshpande et al. 2019; https://github.com/mskilab/dryclean<br>FATHMM Somatic; Shihab et al. 2013; http://fathmm.biocompute.org.uk/<br>Genome Analysis Toolkit (GATK); McKenna et al. 2010; https://software.broadinstitute.org/gatk/<br>Genome Studio Genotyping module v.1.7; This paper; https://doi.org/10.1158/1940-6207.CAPR-13-0289<br>Genome Studio v2010.2; This paper; https://doi.org/10.1158/1940-6207.CAPR-13-0289<br>gGnome; Hadi et al. 2020; https://github.com/mskilab/gGnome

JaBbA; Hadi et al. 2020; https://github.com/mskilab/JaBbA
"LoFreq v2.1.3aNYGC pipeline version 4 (February 12, 2016); Wilm et al. 2012; https://csb5.github.io/lofreq/"
MutationAssessor; Reva et al. 2011; http://mutationassessor.org/r3/
"muTect v1.1.7NYGC pipeline version 4 (February 12, 2016); Cibulskis et al. 2013; https://software.broadinstitute.org/cancer/cga/mutect"
"NBIC-seqNYGC pipeline version 4 (February 12, 2016); Xi et al. 2016; http://compbio.med.harvard.edu/BIC-seq/"
Partek Plug-in v2.13.1; This paper; https://doi.org/10.1158/1940-6207.CAPR-13-0289
pASCAT v2.1; Smith et al. 2018; https://github.com/kuhnerlab/pASCAT
Phylip version 3.695; Felsenstein 1993; http://evolution.genetics.washington.edu/phylip.html
Picard Tools v2.7.0; Broad; http://picard.sourceforge.net
"Pindel v0.2.5NYGC pipeline version 4 (February 12, 2016); Ye et al. 2009; https://github.com/genome/pindel/releases"
Python; Python; https://www.python.org/
R; R Core Team 2020; https://www.R-project.org/
SAS/STAT v9.4
"Scalpel v.0.5.3NYGC pipeline version 4 (February 12, 2016); Narzisi et al. 2014; https://sourceforge.net/projects/scalpel/"
SigProfiler; Alexandrov et al. 2020; https://cancer.sanger.ac.uk/cosmic/signatures/sigprofiler.tt
"snpEff version 4.2(build 2015-12-05); Cingolani et al. 2012; http://snpeff.sourceforge.net/download.html"
snpSift; Cingolani et.al. 2012.; http://snpeff.sourceforge.net/SnpSift.html
"SplazerSNYGC pipeline version 4 (February 12, 2016); Emde et al. 2012; https://omictools.com/splazers-tool"
"Strelka v1.0.14NYGC pipeline version 4 (February 12, 2016); Saunders et al. 2012; https://omictools.com/strelka-tool"
Telomeasure; This paper; https://github.com/nygenome/telomeasure
TITAN; Ha et al. 2014; https://github.com/gavinha/TitanCNA
umap R package; McInnes et al., arXiv:1802.03426; https://cran.r-project.org/web/packages/umap/
VerifyBamId (June 2012); Jun et al. 2012; https://hpc.nih.gov/apps/verifybamid.html

For manuscripts utilizing custom algorithms or software that are central to the research but not yet described in published literature, software must be made available to editors and reviewers. We strongly encourage code deposition in a community repository (e.g. GitHub). See the Nature Portfolio guidelines for submitting code & software for further information.

Data

Policy information about availability of data

All manuscripts must include a data availability statement. This statement should provide the following information, where applicable:

- Accession codes, unique identifiers, or web links for publicly available datasets
- A description of any restrictions on data availability
- For clinical datasets or third party data, please ensure that the statement adheres to our policy

The dataset generated during this study are available at dbGAP:phs001912.v1.p1
https://www.ncbi.nlm.nih.gov/projects/gap/cgi-bin/study.cgi?study_id=phs001912.v1.p1

Field-specific reporting

Please select the one below that is the best fit for your research. If you are not sure, read the appropriate sections before making your selection.

☒ Life sciences ☐ Behavioural & social sciences ☐ Ecological, evolutionary & environmental sciences

For a reference copy of the document with all sections, see nature.com/documents/nr-reporting-summary-flat.pdf

Life sciences study design

All studies must disclose on these points even when the disclosure is negative.

Sample size

The 80 patients with Barrett's esophagus (40 with cancer outcome and 40 without cancer outcome) were selected from a previously published case-cohort study of 248 patients from Li et al, Cancer Prev Res, 2014 Jan;7 PMID: 24253313, in which somatic chromosomal alterations (SCA) had been characterized every two centimeters (cm) in the Barrett's segment. For each cancer outcome case, non-cancer outcome controls were randomly matched on baseline total SCA, age at T1, time between T1 and T2, and gender for the comparisons of the two groups in this study. Four epithelial purified biopsies per patient (160 samples per group) were evaluated. The statistical power was evaluated using previous publication data on mutation, structural variation, and SCA load. For tests for continuous variables, the sample size and statistical power were calculated using the method developed by Diggle et al. (1994) for repeated continuous measurements (four biopsies per patient). For discrete variable such as mutation present or not (0/1) in the two population comparison, the sample size and statistical power was evaluated using method developed by Diggle et al. for repeated discrete variable.

Data exclusions

During patient selection, patients were excluded if they had fewer than two endoscopies (each endoscopy was required to have ≥ two cm segment of BE), and were excluded if they had any of the following interventions: endoscopic mucosal resection, radiofrequency ablation, photodynamic therapy, or esophageal surgical resection for cancer prior to T1.

Replication

The design of the study is a population based study, in which somatic genomes from cancer outcome patients were compared with those from non-cancer outcome patients as two populations. Four independent biopsies were sequenced from each patient to study within patient heterogeneity of somatic genome. The study was carried out using well-established sequencing technology; no individual biopsies were repeatedly tested or replicates analyzed.

Randomization	This was a cancer outcome (case) vs non-cancer outcome (control) study. For each cancer outcome case, non-cancer outcome controls were randomly matched on baseline total SCA, age at T1, time between T1 and T2, and gender for the comparisons of the two groups in this study.
Blinding	All individuals involved in whole genome sequencing and data analysis were blinded to cancer outcome results of patients with the exception of one individual. This individual relabeled samples to blind for patient identity and outcome . After all mutation/indels/SCA calls were completed, then the cancer outcome was revealed to the statistical analysts to perform various quantitative analyses of populations of cancer outcome and non-cancer outcome patients.

Reporting for specific materials, systems and methods

We require information from authors about some types of materials, experimental systems and methods used in many studies. Here, indicate whether each material, system or method listed is relevant to your study. If you are not sure if a list item applies to your research, read the appropriate section before selecting a response.

Materials & experimental systems

n/a	Involved in the study
☒	☐ Antibodies
☒	☐ Eukaryotic cell lines
☒	☐ Palaeontology and archaeology
☒	☐ Animals and other organisms
☐	☒ Human research participants
☒	☐ Clinical data
☒	☐ Dual use research of concern

Methods

n/a	Involved in the study
☒	☐ ChIP-seq
☒	☐ Flow cytometry
☒	☐ MRI-based neuroimaging

Human research participants

Policy information about studies involving human research participants

Population characteristics	Eighty patients with diagnosed Barrett's Esophagus (BE) with longitudinal samples were drawn from a case-cohort study (Li 2014) including 40 cases with BE with non-cancer outcomes "NCO" who did not progress to EA , and 40 controls who progressed to an endoscopically detected, incident EA (cancer outcome, "CO". There were four females and 36 males in each outcome group, CO and NCO. For the NCO group the mean age at first BE diagnosis was 62.77 (range 39.46-79.56), mean age at T1 was 66.14 (range 40.9-87.3), and mean age at T2 was 69.56 (range 42.5-89.9). For the CO group the mean age at first BE diagnosis was 63.71 (range 32.02-80.98), mean age at T1 was 65.52 (range 43-81.8), and mean age for T2 was 68.4 (range 46.5-86.1).
Recruitment	Criteria for selection of Seattle Barrett's Esophagus cohort participants<br>a. Study participants have the premalignant condition, Barrett's esophagus<br>b. Study participants are at least 18 years of age<br>c. Study participants are competent to give informed consent<br>d. Study participants are medically fit to undergo upper endoscopy with biopsy based on the participants' history and physical examination.<br><br>Exclusion Criteria<br>Children and pregnant females were not allowed to participate.<br><br>We aren't aware of any biases in patient recruitment that would influence the outcome of this study.
Ethics oversight	All research participants contributing clinical data and biospecimens to this study provided written informed consent, subject to oversight by the Fred Hutchinson Cancer Research Center IRB Committee D (Reg ID 5619).

Note that full information on the approval of the study protocol must also be provided in the manuscript.
